# Case of Musket-ball in the Bladder

**Published:** 1855-06

**Authors:** James W. Robinson

**Affiliations:** Warfordsburg, Fulton Co., Pa.


*Case of Musket-ball in the Bladder.*

By James W. Robinson, M.D.

Nearly eighteen months since there fell under my observation a case of an anomalous character, which I have ever since desired to make public, but from negligence have suffered it to escape my attention until this late day. Hoping, however, that it may possess sufficient interest to warrant publication even after so great a lapse of time, I transmit to you an imperfect account of the case—imperfect from the fact that it is entirely from memory, having made no written record of the particulars at the time. I was then pursuing the duties of my profession, twenty miles north-west of this place, on the great thoroughfare leading from Chambersburg to Pittsburg. I was called six miles west on that road to see a patient who presented the following history:

He was a man between 30 and 35 years of age, of good physical development, and unquestionably at one time of fine constitution. Two years previous to the time he fell under my care, he was engaged in trading, in the State of Texas, whither he had migrated from Cincinnati, his former home. While pursuing his avocation in that State, he received a shot from a musket ball, which entered the depression formed by the glutei muscles on the outside of the hip, and passing through, behind the femur, lodged, he could not tell where. He lay for some weeks from the effects of the wound, without any medical or surgical treatment, and finally recovered sufficiently to return to Cincinnati. Here he entered the hospital, where he informed me that he suffered for some time from a vesico-rectal fistula, which was eventually cured by repeated cauterization; also, that either the wadding of the gun or a portion of his clothing driven before the bullet, had been removed by an operation, from the cavity of the bladder. During all this period, and up to the time at which I met with him, he could only pass his urine with much pain, by aid of a gum catheter which he had learned to insert himself, and carried constantly with him; the urine always being loaded with more or less purulent matter. Life becoming a burden to him in this situation, and his means of subsistence being reduced to an extremity, and not satisfied with what had been done for him in the Cincinnati hospital, he made his way to a similar institution in Pittsburg, at which place nothing more in the way of an operation was attempted, and no prospect of relief afforded him. He entertained some vague notion that an operation might be performed which would relieve his condition, but had no conception of the nature or rationale of it. Hence he conceived the idea, no doubt from the advice of some other person, of endeavoring to seek his way from Pittsburg to the Pennsylvania Hospital at Philadelphia, for the purpose of presenting his case to the surgeons of that institution. With that in view, his last hope of earthly comfort, he started from Pittsburg in his critical state of health, on foot and without money.

Buoyed up with the prospect of a relief of his sufferings, he travelled on at a slow pace, in the month of October, the weather cold and his clothing light. On one of the days in the latter part of that month, there came on a heavy storm of snow and rain, lasting throughout the day, and covering the ground to a considerable depth. During the whole of this day he wandered over long and steep hills, drenched with rain and chilled with snow, and halted at night at a tavern 116 miles east of Pittsburg, sick and exhausted, his day's journey having sown within him the seeds of death. After lying sick for a number of days without any medical treatment or attendance, I was called upon to visit him. I found him in a miserable condition, complaining of intense pain over the left kidney and in the region of the bladder, and passing, by means of the catheter, considerable quantities of urine heavily charged with pus, with much suffering during the evacuation of the bladder. He had labored under considerable febrile reaction, which had now assumed a real typhous type. He had for a length of time, that is in all probability ever since he received the wound, suffered from chronic inflammation of the bladder, which was forced into a more active state by his exposure to the inclement weather, while there was superadded evident acute inflammation of the left kidney. His tongue had assumed the dark brown or blackish aspect, so characteristic of his low typhous condition, with all the other symptoms corresponding. In the way of treatment, my efforts were directed towards effecting a mercurial impression, both by internal and external use, but without avail. Demulcents and stimulants, ammonia and wine, and finally brandy and the application of powerful sinapisms and other medicines, and applications which have escaped my memory, and the recollection of which for our present purpose would be of little importance, were also used. He seemed to rally slightly sometimes, but lingering on for near ten days, he sank and died under copious hemorrhage from the bladder and bowels simultaneously. Two hours after death I proceeded to make a postmortem examination, which was, of necessity, very imperfect from the want of assistance, scarcely being able to procure the services of any one to furnish me a sufficiency of light (it being in the night) from the horror people out of the profession pretend to have for such operations. The examination, however, was sufficiently minute to invalidate my diagnosis, and to reveal a very remarkable case. The left kidney I found to be a mass of suppuration. The bladder was much indurated, and its coats thickened to an incredible extent. Laying open its walls I found within its cavity a musket ball of the largest size, with a calculus three or four times the size of the bullet attached to its side. I have both the ball and the calculus in my possession, though they have become detached by violence. The ball weighs more than an ounce, and is the largest I ever saw. The calculus measures more than an inch in every dimension, except one, being a little flattened and of a cylindrical shape.

The singularity of the case is in the fact that a large musket ball, with a portion of clothing, was driven entirely through the fleshy part of the hip within the cavity of the pelvis, and finding a lodgement in the bladder, allowed it to heal over it, and that it remained there for two years without causing disturbance inconsistent with life. Also, that after the first shock of the injury he underwent a partial recovery without any treatment. It is also very singular that he came through the hands of a number of surgeons who failed even to detect the presence of these foreign bodies in the bladder. I regret very much that he did not reach the Pennsylvania Hospital in as good health as he left Pittsburg, then able to make a long journey on foot in bad weather. I am very sure the able surgeons of that veteran institution would not have mistaken his case, but would have unhesitatingly submitted him to the operation of lithotomy, with every prospect of saving his life and restoring his health.

I regret that my recollection and a more minute examination of the case do not enable me to give an account marked with that exactness and clearness of detail which must be a characteristic of every good clinical or post-mortem report.

*Warfordsburg, Fulton Co., Pa., April 17th,* 1855.

